# Prevalence of Metabolic Syndrome among nursing personnel and its association with occupational stress, anxiety and depression^1^

**DOI:** 10.1590/0104-1169.0383.2573

**Published:** 2015-07-03

**Authors:** Renata Perfeito Ribeiro, Maria Helena Palucci Marziale, Julia Trevisan Martins, Patrícia Helena Vivan Ribeiro, Maria Lucia do Carmo Cruz Robazzi, José Carlos Dalmas

**Affiliations:** 2 PhD, Adjunct Professor, Departamento de Enfermagem, Universidade Estadual de Londrina, Londrina, PR, Brazil; 3 PhD, Full Professor, Escola de Enfermagem de Ribeirão Preto, Universidade de São Paulo, PAHO/WHO Collaborating Centre for Nursing Research Development, Ribeirão Preto, SP, Brazil; 4 PhD, RN, Clínica Odontológica Universitária, Universidade Estadual de Londrina, Londrina, PR, Brazil

**Keywords:** Occupational Health, Obesity, Metabolism, Burnout, Professional, Anxiety, Depression, Nursing

## Abstract

**OBJECTIVE:**

to identify the prevalence of Metabolic Syndrome among nursing personnel, and its association with occupational stress, anxiety and depression.

**METHOD:**

a descriptive correlational study undertaken with 226 nursing personnel from a teaching hospital. Data collection was undertaken through application of the Job Stress Scale, the Hospital Anxiety and Depression Scale and a sociodemographic questionnaire, with variables of Metabolic Syndrome. Univariate analyses and Chi-squared and Pearson tests were used for correlation between the variables, with a level of significance of 5%.

**RESULTS:**

86 (38.1%) workers presented Metabolic Syndrome, of whom 183 (81.1%) were female, and 43 (19.9%) male, aged between 23 and 66 years old. In relation to anxiety and depression, 154 (68.1%) presented anxiety, with 48 (31.2%) also presenting Metabolic Syndrome; 185 (81.8%) presented depression, of whom 62 (33.5%) also had Metabolic Syndrome. It was ascertained that 61 (27.0%) workers presented stress and that of these, 14 (22.9%) presented Metabolic Syndrome.

**CONCLUSION:**

a correlation was observed between the variables of anxiety and Metabolic Syndrome and stress and Metabolic Syndrome, there being no correlation between the variables of depression and Metabolic Syndrome.

## Introduction

Contemporary political, social, economic and cultural changes have transformed humanity's relationship with work. The new forms of the organizational process of work incessantly undergo increasingly complex, profoundly sophisticated changes, which are reflected in the workers' health^1^.

The profile of healthcare workers' morbidity and mortality is characterized by a coexistence with harm, with work accidents and with occupational illnesses, which are directly related to the specific work conditions and how the work is organized, added to by the illnesses common to the population in general.

Among the common illnesses in the population, emphasis is placed upon Metabolic Syndrome (MetS). This illness, related to the endocrine system, affects a large number of people worldwide and severely influences these peoples' quality of life and work.

MetS is a clinical entity with metabolic and hormonal changes, characterized by abdominal obesity, insulin resistance, Arterial Hypertension (AH) and dyslipidemia^2^. It is a complex disorder, represented by a set of cardiovascular risk factors, usually related to the deposition of fat and resistance to insulin. From the epidemiological point of view, its importance is highlighted, as it is responsible for the increase - by up to 2.5 times - of mortality related to cardiovascular causes in Brazil^3^.

There is not yet any strong scientific evidence proving the direct relationship between MetS and work activity^4^, however, it is believed that the nursing team's working conditions can contribute to its development, due to erroneous eating habits, caused by irregular mealtimes, night work and shift work, and the physical and psychological burdens related to the imminent risk of the patient's death and to the care for the patient's family members - as well as to the interpersonal relationship within the health team which can also contribute to the development of stress.

Occupational stress is constituted by the association between the various symptoms presented by the organism, and can trigger physical and mental illnesses. Workers with chronic stress have double the chances of developing MetS^5^, sleep disorders, chronic fatigue, diabetes and Burnout syndrome^6^. The complexity of the relationships between people, the inadequate planning of human and material resources, and the nursing work environment are also factors which contribute to the emergence of stress and anxiety^7^. Authors assert that there is a relationship between MetS, anxiety and depression^8^.

In one study undertaken in London, the Whitehall II study, which studied chronic stress among British workers, an association was found between chronic stress at work and presence of MetS^9^. In Brazil, there are studies which evidence the relationship between arterial hypertension and stress at work^10^ and an association between obesity and stress in production sectors^4^, but there is not yet evidence of the association of MetS with stress, anxiety and depression among workers of the nursing team.

One study on the factors predisposing to MetS emphasized the importance of undertaking further studies on the chronic stress and the development of MetS in unhealthy workplaces, as occurs with workers from the area of health care who work in hospitals^5^.

This study was proposed due to the gaps in the scientific knowledge relating to the correlation of the variables of MetS, anxiety, depression and stress among nursing workers.

The general objective of the study was to identify the prevalence of Metabolic Syndrome among nursing workers, and its association with occupational stress, anxiety and depression.

## Method

A descriptive, correlational and transversal study was undertaken. The Demands-Control Model was adopted as the theoretical framework^10^^-^ 11, for analysis of the relationship between stress and work, in the conceptual assumptions regarding the factors which predispose to MetS^5^ and in the conception regarding work-related anxiety and depression^12^.

**Figure 1:**
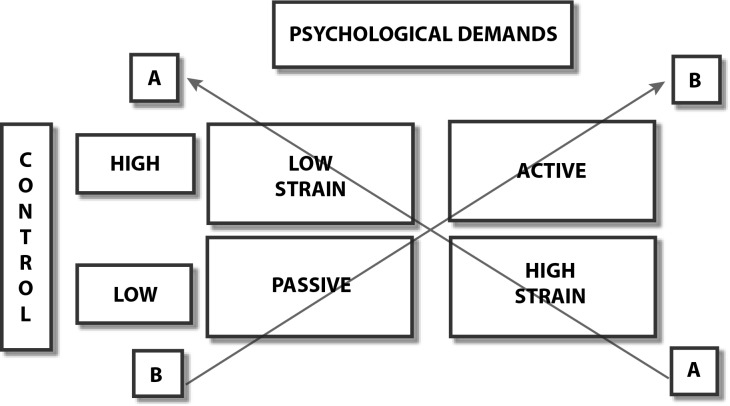
Diagram of the control, demand and social support relationship, as proposed by Theorell and Karasek (1996).

 Figure 1 diagrams the control, demand and social support relationship, presented by the study participants.

The study was undertaken in a teaching hospital in the State of Paraná (PR), Brazil. The population was composed of the nursing team, made up of 704 workers, of whom 133 were male and 571 female, distributed in the following categories: nurses, nursing technicians, and auxiliary nurses.

The inclusion criteria for the sample were: to have undertaken the regular health tests between August 2011 and August 2012, to have had a permanent contract with the institution for over two years, and to work in direct patient care. Those workers who were retiring, on leave, or on holiday were excluded.

The study sample was calculated taking into account a level of significance of 5% and a margin of error of 0.05. Based on the sample calculation, the study was undertaken with 226 people, this being 32.1% of the total population of the nursing workers, of whom 183 (81.1%) were female and 43 (18.9%) were male.

For data collection, the following instruments were used: sociodemographic, occupational and health characteristics of the workers, the Job Stress Scale (JSS) adapted and validated to Portuguese^10^ and the Hospital Anxiety and Depression Scale (HADS)^12^. Data relating to blood biochemistry (total cholesterol, High Density Lipoproteins (HDL) and Low Density Lipoproteins (LDL), Triglycerides (TGL) and fasting glycemia) were obtained from the nursing workers' medical records.

Following application of the instruments, data relating to the participants' vital signs (blood pressure and cardiac frequency) was checked.

The results were interpreted according to the I Brazilian Directives for Diagnosis and Treatment of Metabolic Syndrome (Brazilian Society of Cardiology, 2005), which adopted in full what was established by the NCEP-ATP III, whose criteria require the finding of three or more of the following components for the diagnosis of MetS: abdominal circumference: > 102 cm for men and > 88 cm for women; blood pressure: ≥ 130/85mmHg; fasting glycemia: ≥ 110mg/dL; triglycerides: ≥ 150mg/dL; HDL-cholesterol: < 40mg/dL for men and < 50mg/dL for women, as well as the use of medications for controlling arterial hypertension and anti-hyperlipidemic agents.

The data were entered in an Excel 2000^(r)^ spreadsheet and processed using the Statistical Package for Social Sciences (SPSS) software, version 15.0.

For the characterization of the correlation of stress, anxiety and depression with MetS, as well as the presentation of the results in absolute and percentage values, and ordinal and nominal variables, the Chi-squared correlation test and Pearson test were undertaken for comparison among the variables, determining the statistical correlation between the same, considering a level of significance for the study of 5% (α=0.05).

In order to characterize the participants' state of health and the correlation of the stress, anxiety and depression with MetS, the descriptive analysis of the quantitative variables was undertaken; in order to ascertain the correlations between the variables, the Chi-squared test and Pearson test were applied, considering a level of significance of 5% (α=0.05).

The project was approved by the Committee for Ethics in Research with Human Beings (CAAE N. 0218.0.268.153-09), following all the recommendations of the National Committee for Ethics in Research.

## Results

In accordance with the data presented in Table 1, among the 226 participants in this study, the majority were female (75.8%), aged between 23 and 66 years old, and MetS was present in the 38.1% of the workers.

**Table 1.** Distribution of nursing workers in the Teaching Hospital, by age, sex and presence of Metabolic Syndrome. Londrina, PR, Brazil, 2012

Variable		N (%)	Interval
Age		226 (100.0)	23 - 66
Sex			
	Female	171 (75.8)	
	Male	55 (24.2)	
Metabolic Syndrome			
	Yes	86 (38.1)	
	No	140 (61.9)	

According to the data presented in Table 2, of the 86 (38.0%) workers who had MetS, the mean age was 45 years old, with standard deviation of (SD±9.13) years.

**Table 2.** Distribution of the age of the workers of the nursing team at the Teaching Hospital, by presence of Metabolic Syndrome. Londrina, PR, Brazil, 2012

Age	Metabolic Syndrome		Total (%)
	Yes (%)	No (%)	
23 - 40 years old	24 (27.9)	53 (37.9)	77 (100.0)
41 - 50 years old	36 (41.9)	72 (51.4)	108 (100.0)
51 - 66 years old	26 (30.2)	15 (10.7)	41(100.0)

Table 3 presents data referent to the presence of MetS in the workers of the nursing team and to the presence of anxiety, depression and stress.

**Table 3.** Distribution of Metabolic Syndrome in nursing staff of the Teaching Hospital, and the presence of anxiety, depression and stress. Londrina, PR, Brazil, 2012

Metabolic Syndrome	Anxiety	Depression	Stress
Yes	48 (31.2%)	62 (33.5%)	14 (22.9%)
No	106 (68.8%)	123 (66.5%)	47 (77.1%)

A correlation was observed (p= 0.022) between the variables of anxiety and MetS, stress and MetS ( p= 0.008), and an absence of correlation (p= 0.052) was observed between the variables of depression and MetS.

## Discussion

The sociodemographic characteristics of the workers in the present study are similar to those found in other studies undertaken, both in Brazil and internationally^13^^-^ 16, with a predominance of female participants.

In one study undertaken^17^ with the objective of assessing the association between psychiatric disorders and MetS, the prevalence of MetS was greater among women than among men, both with depression. This fact may be related to the women's stressful lifestyle, such as, for example, feelings of anger and hostility which correlate significantly with hyperinsulinemia, hyperglycemia, dyslipidemia, hypertension and central obesity, confirming that psychological risk factors affect the development of metabolic syndrome^18^. In relation to the workers' age, it varied between 23 and 66 years old; equivalent data were identified in a study which evaluated the quality of life in the work of nursing professionals in the surgical center^16^.

Among the 86 (38.1%) workers with MetS, the mean age was 45 years old, with standard deviation of (SD±9.13) years. These data are different from another study^19^ which aimed to ascertain the prevalence of, and factors associated with, Mets, in which the mean age of the participants with MetS was 58.3 years old. These data confirm those of the present study, that the workers present MetS at an earlier age.

Academics confirm that MetS can be a predisposing factor for the development of depression^20^. Furthermore, authors show that individuals who present symptoms of depression have high levels of triglycerides, an increase in abdominal circumference, and high lipoprotein density^21^.

Depression can affect from 4% to 7% of the general population, being configured as a very frequent disorder^22^. Depression's link with other illnesses, including MetS, has been investigated, bringing new information, in which the two pathologies share the same symptoms and consequences, such as increase in total body mass, diabetes, insulin resistance and increase of mortality from cardiovascular diseases^22^.

Anxiety and depression can predispose to MetS, as behavioral disorders of anxiety and depression often occur simultaneously and are linked to higher cardiometabolic risk of acute cardiovascular events^8^.

The results obtained in this study showed the correlation between anxiety and the presence of MetS, with no correlation between the presence of MetS and depression.

A study undertaken in Australia, with the objective of identifying, in the population in general, the relationship between diabetes, depression and cardiovascular disease, and which used the same instrument for verification as the present study, the HADS, ascertained that MetS has a significant correlation with depression, but not with anxiety^23^.

There is a relationship between MetS and depression, as depression is linked to the increase in cortisol in the blood, increasing glucose intolerance, blood pressure and weight gain^22^. These same authors state that the serotonergic system may be involved in the association between MetS and depression, in which the reduction in the function of this system results in greater ingestion of carbohydrates^22^. The hypothesis of hyperactivation of the hypothalamic-pituitary-adrenal axis (HPA) has been the most accepted as a response to the link between mental disorders and MetS^22^. In depression, the hyperactivity of the HPA axis may be a more consistent biochemical finding for a correlation between depression and MetS^24^.

A depressed person has great difficulty in undertaking physical exercise and a lack of desire to undertake a healthy diet, presenting irregular eating habits, increasing her susceptibility to obesity, cholesterol and triglycerides and, thus, glucose intolerance^22^, favoring the development of MetS.

The present study's results indicate that there is a correlation (p= 0.022) between anxiety and MetS, and an absence of correlation (p= 0.052) between depression and MetS.

One study undertaken with Japanese workers, aiming to verify the association between MetS, depression and anxiety, identified a correlation between MetS, anxiety and depression; 12.2% of the workers presented MetS, 7.6% presented depression, and 14% presented anxiety^20^.

Remaining in this study, a correlation (p=0.008) was observed between the variables of stress and MetS. In relation to stress, these results may be linked to the unsatisfactory conditions of the nursing team's work process, such as lack of qualified personnel, lack of material resources, a constant search for improvements in the light of technological and scientific advances, relationships within the team, high rotation within shifts and departments, high demands from patients, a high number of critically-ill patients, lack of, and poor functioning of, equipment, the relationship with patients' family members^25^ and low pay; these being factors which hinder the adoption of measures promoting a healthy life, such as undertaking physical exercise, eating appropriately and recreational activities, leading to the appearance of MetS.

Although this study's objectives have been achieved, limitations were observed, as the transversal design did not allow the generalization of the findings to other contexts. It is necessary to undertake research with methodological designs which allow the monitoring of workers, for a possible definition of cause and effect among the variables of MetS, anxiety, depression and stress.

This study's results, however, contribute to advances in scientific knowledge in the area of Occupational Health and for Nursing, given that it is possible to use its results in programs for prevention of illness at work in hospital institutions, and that it supports the undertaking of future studies.

## Conclusion

This research's results evidenced the correlation between the variables of Metabolic Syndrome and anxiety, and Metabolic Syndrome and stress, among nursing workers.

The study provides support for future research, and draws attention to the need for greater attention to these peoples' health, and the adoption of strategies for promoting occupational health.

## References

[1] Ribeiro RP, Martins JT, Marziale MHP, Robazzi MLCC (2012). O adoecer pelo trabalho na enfermagem: uma revisão
integrativa. Rev Esc Enferm USP..

[2] Franke AL, Suplicy H (2007). Síndrome metabólica. Rev Bras Medicina..

[3] Sociedade Brasileira de, Cardiologia (2005). I Diretriz brasileira de diagnóstico e tratamento da síndrome
metabólica. Arquivos Brasileiros de Cardiologia.

[4] Ribeiro RP, Ribeiro PHV, Marziale MHP, Martins MB, Santos MR (2011). Obesity and stress among workers from different sectors of production:
an integrative review. Acta Paul Enferm..

[5] Chandola T, Brunner E, Marmot MG (2006). Chronic stress at work and the metabolic syndrome: prospective
study. BMJ..

[6] Limongi-França AC, Rodrigues AL (2005). Stress e trabalho: uma abordagem psicossomática.

[7] Lindhol MM (2006). Working conditions, psychosocial resources and work stress in nurses
and physicians in chief managers' positions. J Nurs Manag..

[8] Rosolová H, Podlipný J (2009). Anxious-depressive disorders and metabolic syndrome. Vnitrní lékarství..

[9] Marmot MG, Brunner E (2005). cohort profile: the Whitehall II study. Int Epidemiol..

[10] Alves MGM, Chor D, Faerstein T, Lopes CS, Werneck GL (2004). Versão resumida da "job stress scale": adaptação para o
português. Rev Saúde Pública..

[11] Theorell T, Karasek RA (1996). Current issues relating to psychosocial job strain and cardiovascular
disease research. J Occup Health Psychol..

[12] Botega NJ, Bio MR, Zomignani MA, Garcia C, Pereira WAB (1995). Transtornos de humor em enfermaria de clínica médica e validação da
escala de medida (HAD) de ansiedade e depressão. Rev Saúde Pública..

[13] Guerrer FJL, Bianchi ERF (2008). Caracterização do estresse nos enfermeiros de unidades de terapia
intensiva. Rev Esc Enferm USP..

[14] Krogstad U, Hofoss D, Veenstra M, Hjortdahl P (2006). Predictors of job satisfaction among doctors, nurses and auxiliaries
in Norwegian hospitals: relevance for micro unit culture. Human Resources for Health..

[15] Urbanetto JS, Silva PC, Hoffmeister E, Negri BS, Pinheiro da Costa BE, Poli de Figueiredo CE (2011). Workplace stress in nursing workers from an emergency hospital: Job
Stress Scale analysis. Rev. Latino-Am. Enfermagem..

[16] Schmidt DRC, Dantas RAS, Marziale MHP, Laus AM (2009). Estresse ocupacional entre profissionais de enfermagem do bloco
cirúrgico. Texto Contexto Enferm..

[17] Teixeira PJ, Rocha FP (2007). Associação entre síndrome metabólica e transtornos
mentais. Rev Psiquiatr Clín..

[18] Räikkönen K, Mattheus KA, Kuller LH (2002). The relationship between psychological risk attributes and the
metabolic syndrome in healthy women: antecedent or consequence?. Metabolism..

[19] Franco GPP, Scala LCN, Alves CJ, França GVA, Cassanelli T, Jardim PCBV (2009). Metabolic syndrome in patients with high blood pressure in Cuiabá -
Mato Grosso State: prevalence and associated factors. Arq Bras Cardiol..

[20] Takeuchi T, Nakao M, Nomura K, Inoue1 M, Tsurugano S, Shinozaki Y (2009). Association of the metabolic syndrome with depression and anxiety in
Japanese men: a 1-year cohort study. Diabetes Metab Res Rev..

[21] East C, Willis BL, Barlow CE, Grannemann BD, FitzGerald SJ, DeFina LF (2010). Depressive symptoms and metabolic syndrome in preventive healthcare:
the Cooper Center longitudinal study. Metab Syndr Relat Disord..

[22] Ballone GJ, Ximenes BAA (2008). Obesidade, síndrome metabólica e depressão.

[23] Dunbar JA, Reddy P, Davis-Lameloise N, Philpot B, Laatikainen T, Kilkkinen A (2008). Depression: an important comorbidity with metabolic syndrome in a
general population. Diabetes Care..

[24] Ramasubbu R (2002). Insuline resistance: a metabolic link between depressive disorder and
atherosclerotic vascular diseases. Med Hypotheses..

[25] Gomes GC, Lunardi WD, Erdmann AL (2006). O sofrimento psíquico em trabalhadores de UTI interferindo no seu modo
de viver a enfermagem. Rev Enferm UERJ..

